# The ferroptosis landscape in acute myeloid leukemia

**DOI:** 10.18632/aging.205257

**Published:** 2023-11-29

**Authors:** Zhixin Ma, Wenle Ye, Xin Huang, Xia Li, Fenglin Li, Xiangjie Lin, Chao Hu, Jinghan Wang, Jie Jin, Bo Zhu, Jiansong Huang

**Affiliations:** 1 Clinical Prenatal Diagnosis Center, Key Laboratory of Reproductive Genetics, Women's Hospital, Zhejiang University School of Medicine, Hangzhou, Zhejiang, China; 2 Department of Hematology, Key Laboratory of Hematologic Malignancies, Diagnosis and Treatment, The First Affiliated Hospital, Zhejiang University School of Medicine, Hangzhou, Zhejiang, China

**Keywords:** acute myeloid leukemia, ferroptosis, glutathione peroxidases 4, apoptosis-inducing factor mitochondria-associated 2, chemotherapy resistant

## Abstract

Ferroptosis induction through the suppression of glutathione peroxidase 4 (GPX4) and apoptosis-inducing factor mitochondria-associated 2 (AIFM2) has proven to be an effective approach in eliminating chemotherapy-resistant cells of various types. However, a comprehensive understanding of the roles of GPX4 and AIFM2 in acute myeloid leukemia (AML) has not yet been achieved. Using cBioPortal, DepMap, GEPIA, Metascape, and ONCOMINE, we compared the transcriptional expression, survival data, gene mutation, methylation, and network analyses of GPX4- and AIFM2-associated signaling pathways in AML. The results revealed that high expression levels of GPX4 and AIFM2 are associated with an adverse prognosis for AML patients. Overexpression of AIFM2 correlated with elevated mutation frequencies in NPM1 and DNMT3A. GPX4 upregulation modulated the following pathways: GO:0045333, cellular respiration; R-HSA-5389840, mitochondrial translation elongation; GO:0009060, aerobic respiration; R-HSA-9609507, protein localization; and R-HSA-8953854, metabolism of RNA. On the other hand, the overexpression of AIFM2 influenced the following processes: GO:0048704, embryonic skeletal system morphogenesis; GO:0021546, rhombomere development; GO:0009954, proximal/distal pattern formation; and GO:0048732, gland development. This study identifies the high expression of GPX4 and AIFM2 as novel biomarkers predicting a poor prognosis for AML patients. Furthermore, ferroptosis induction may improve the stratified treatment of AML.

## INTRODUCTION

Acute myeloid leukemia (AML) comprises a group of heterogeneous hematologic malignancies characterized by an increased number of cytogenetic and molecular abnormalities, imposing a profound burden on affected individuals worldwide [1, 2]. Despite advancements in AML treatment, such as risk stratification, combination chemotherapy, and stem cell transplantation, the overall survival rate remains unsatisfactory [3, 4]. While the genomic landscape of AML has been extensively characterized, revealing numerous potential therapeutic targets, but how to effectively kill AML cells while leaving healthy cells uninjured remains a fundamental challenge.

Ferroptosis induction, an iron-dependent form of necrotic cell death triggered by excessive peroxidation of polyunsaturated fatty acids (PUFAs), is being explored as an alternative approach to eradicate apoptosis-resistant cancer cells [5, 6]. Ferroptosis is identified with hallmarks that are distinct from those of apoptosis; it is characterized by excessive iron-catalyzed peroxidation of PUFA-containing phospholipids (PLs) and is extensive in the mammalian cell membrane [7–9]. PL peroxidation is primarily mediated by reactive oxygen species (ROS) and the activity of the lipoxygenase (LOX) family. Failure to initiate protective mechanisms against peroxidation-induced membrane rupture leads to the induction of ferroptosis [10, 11]. Consequently, ferroptosis is associated with a series of metabolic disorders, including those involving ROS, iron, and PLs. Abnormal genes and pathways related to the metabolism of iron, energy, oxidative stress, and lipid peroxidation may potentially modify cell sensitivity to ferroptosis.

Phospholipid (PL) detoxification is typically regulated by the glutathione peroxidase (GPX) family [12]. To date, GPX4 stands out as the sole GPX responsible for safeguarding membranes against peroxidative damage, and GPX4 functions in a glutathione (GSH)-dependent manner to reduce lipid hydroperoxide levels [13, 14]. Depletion of intracellular GSH or the inhibition of GSH synthesis can indirectly deactivate GPX4, leading to the induction of ferroptosis [15].

Beyond GPX4, ferroptosis suppressor protein 1 (FSP1), also known as apoptosis-inducing factor mitochondria-associated 2 (AIFM2), has recently been identified as another player in suppressing ferroptosis, particularly in the context of GPX4 knockout. AIFM2 protects against ferroptosis through a glutathione-independent mechanism mediated by ubiquinone (CoQ_10_). This compound neutralizes lipid peroxyl radicals, moderating PL peroxidation. AIFM2 catalyzes the production of CoQ_10_ via NAD(P)H. Therefore, the AIFM2-CoQ_10_-NAD(P)H pathway constitutes a parallel system that coordinates with GPX4 and glutathione to maintain the homeostasis of phospholipid peroxidation and ferroptosis [16].

Studies have demonstrated that inducing ferroptosis may effectively eliminate chemotherapy-resistant cancer cells in ovarian cancer, breast cancer, and lung cancer, highlighting the potential of ferroptosis induction as a novel anticancer therapy [17, 18]. Consequently, various ferroptosis inducers (FIs) have gained approval from the Food and Drug Administration (FDA) for clinical use [19]. However, the evaluation of the ferroptosis landscape has not been conducted in the context of AML. In this study, we utilized widely accepted public databases to assess ferroptosis resistance in AML, revealing a relationship between ferroptosis regulatory genes and the pathogenesis and progression of AML.

## RESULTS

### Transcriptional levels of GPX4 and AIFM2 in patients with AML

**Figure 1:**
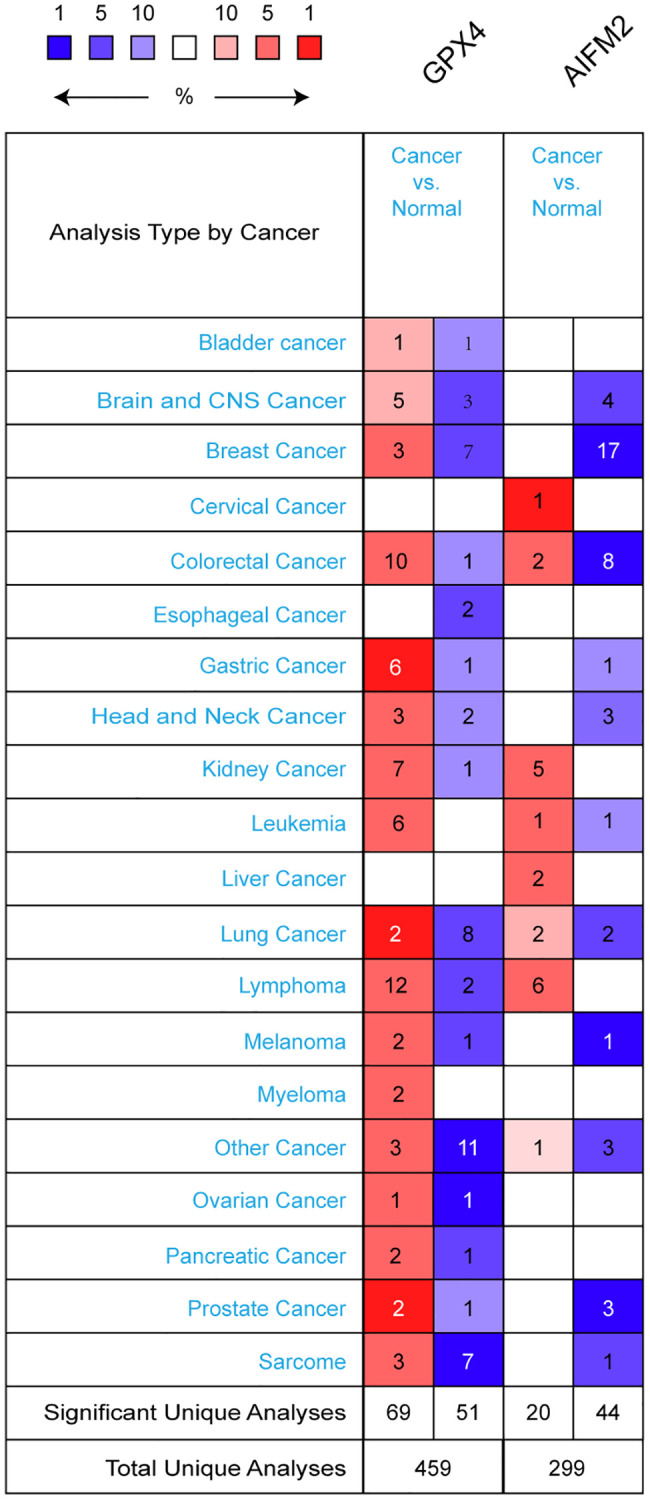
The transcription levels of GPX4 and AIFM2 in different types of cancers.

GPX4 and AIFM2 are genes identified in the human genome. To compare their transcriptional levels in cancer and normal controls, we utilized the ONCOMINE database (Figure 1). ONCOMINE analysis revealed an upregulation of GPX4 and AIFM2 mRNA expression in patients with AML (Table 1). Specifically, GPX4 showed upregulation in 3 datasets, while AIFM2 was overexpressed in 1 dataset. Haferlach’s research indicated a 1.334-fold increase in GPX4 mRNA in AML [20]. Andersson and colleagues reported a 1.74-fold elevation of GPX4 in AML [21], and Valk identified GPX4 with a fold change of 1.257 in AML [22]. Regarding AIFM2 mRNA expression, Haferlach found a 1.069-fold increase in AML [20].

**Table 1:** The significant changes of GPX4 and AIFM2 expression in transcription level between different types of leukemia and normal control.

**GENE**	**Type of leukemia versus normal samples**	**Fold change**	***P* value**	***T* test**	**References**
GPX4	Acute myeloid leukemia versus normal	1.334	3.91E-18	10.639	Haferlach [20]
	Acute myeloid leukemia versus normal	1.257	4.00E-03	NA	Valk [22]
	Acute myeloid leukemia versus normal	1.74	1.73E-04	4.865	Andersson [21]
AIFM2	Acute myeloid leukemia versus normal	1.069	1.02E-04	3.812	Haferlach [20]

To deepen our understanding of ferroptosis in AML, we analyzed GPX4 and AIFM2 transcriptional levels in AML FAB subtypes. In Gutierrez’s dataset, GPX4 was upregulated in M1, M4E0, and M5 with fold changes of 1.47, 1.861, and 1.297, respectively [23]. Bullinger’s data showed fold changes of 1.643 and 1.392 in the M3 and M5 subtypes [24]. Debernardi’s dataset revealed GPX4 overexpression with a fold change of 1.268 in the M4E0 subtype [25]. Notably, AIFM2 was significantly overexpressed in the M5 and M6 subtypes, with no obvious change in the entire AML cohort. Heuser’s dataset indicated AIFM2 increases with a fold change of 2.918 in M5, as well as fold changes of 1.946 and 1.419 in a TCGA study and Metzeler’s dataset [26, 27]. In Wouters’ dataset, AIFM2 increased by 2.066-fold for the M6 subtype [28] (Table 2).

**Table 2:** The significant changes of GPX4 and AIFM2 expression in FAB subtype of AML.

**GENE**	**FAB subtype**	**Fold change**	***P* value**	***T* test**	**References**
GPX4	M0	1.047	0.004	2.718	TCGA Leukemia 2
	M5	1.059	0.029	1.979	TCGA Leukemia 2
	M5	1.392	7.05E-04	3.536	Bullinger [24]
	M3	1.643	0.001	3.549	Bullinger [24]
	M4E0	1.268	0.005	3.021	Debernardi [25]
	M4E0	1.861	0.038	2.2	Gutierrez [23]
	M1	1.47	0.035	2.116	Gutierrez [23]
	M5	1.297	0.042	1.906	Gutierrez [23]
	M4	1.106	0.047	1.693	Valk [22]
	M5	1.197	0.008	2.54	Metzeler [27]
	M5	1.139	1.87E-04	3.624	Wouters [28]
	M1	1.153	0.025	1.991	TCGA Leukemia
AIFM2	M5	2.918	0.002	3.291	Heuser [26]
	M5	1.946	1.12E-05	4.753	TCGA Leukemia
	M6	2.066	0.043	2.045	Wouters [28]
	M5	1.122	2.03E-04	3.611	Wouters [28]
	M5	1.419	0.028	2.426	Metzeler [27]
	M4	1.151	4.90E-02	1.78	Metzeler [27]

### GPX4 and AIFM2 translational factor expression in leukemia cell lines and AML patients

**Figure 2:**
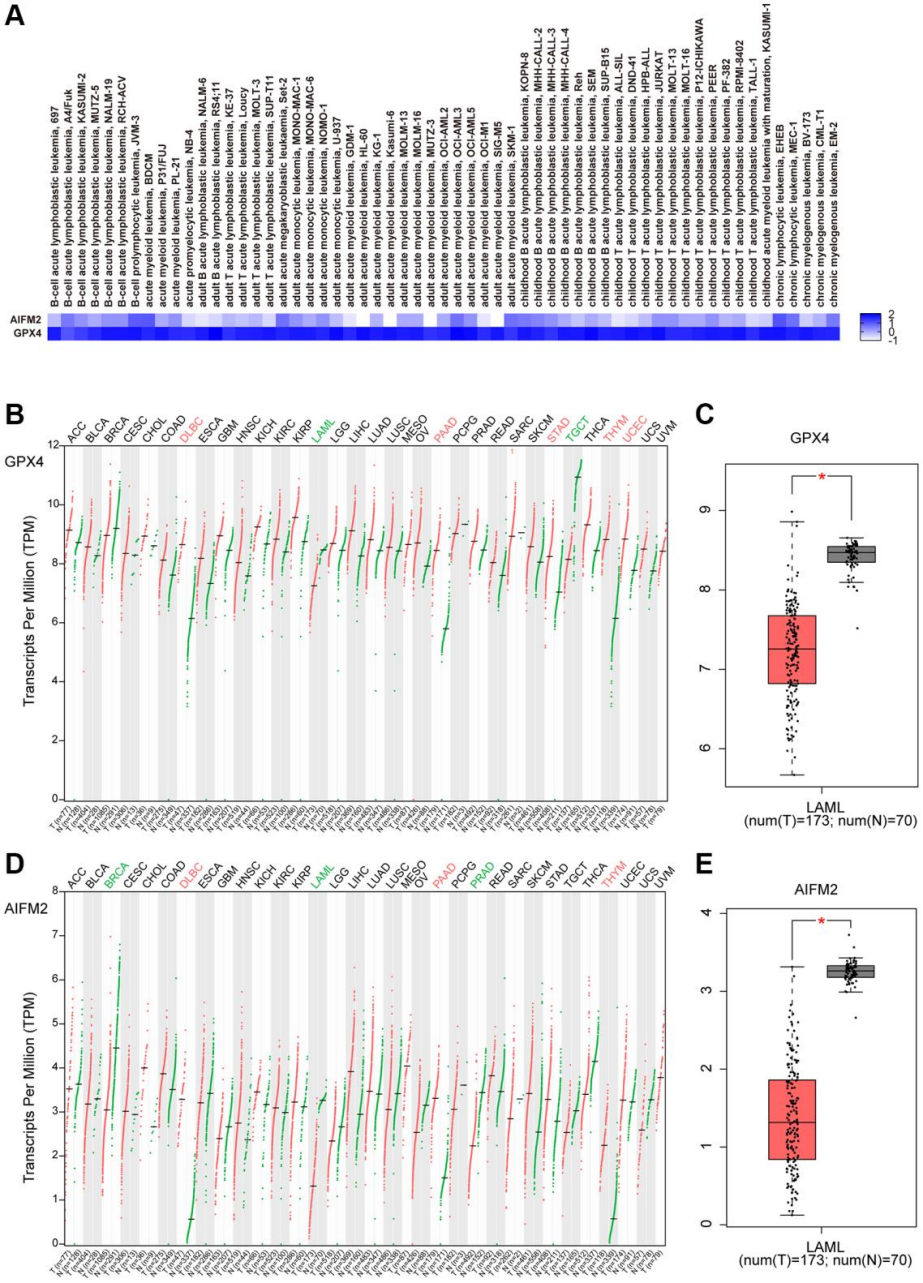
The expression of GPX4 and AIFM2 in leukemia cell lines (**A**) and cancer types (**B**–**E**).

By using the EMBL-EBI bioinformatics website, GPX4 and AIFM2 expression levels were analyzed in leukemia cell lines. The results showed that GPX4 is generally highly expressed in leukemia cell lines. AIFM2 was highly expressed in AML cell lines, including SKIM-1, OCI-AML2, OCI-AML3, OCI-AML5, THP-1, PL-21, and BDCM cells (Figure 2A). Interestingly, the GEPIA assay revealed that both GPX4 and AIFM2 expression levels in the GTEx database of healthy people were higher than those of AML patients in the TCGA database (Figure 2B–2E).

### The dependency of leukemia cells on GPX4 and AIFM2

**Figure 3:**
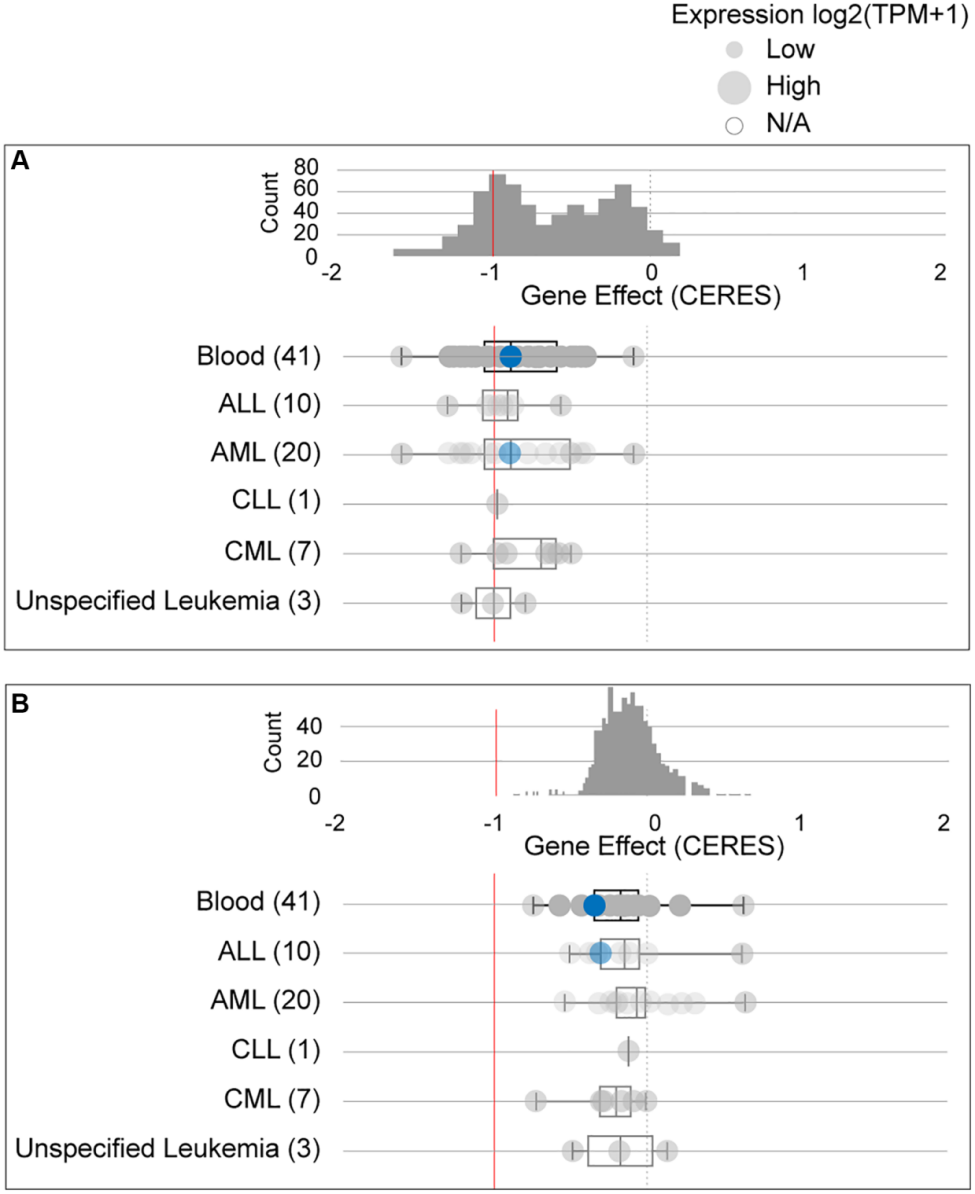
The dependency of leukemia cells on GPX4 (**A**) and AIFM2 (**B**). The blue circle represents the SHI-1 AML cell line with nonconserved gene mutations. A score < 0 means that the selected cell line is more likely to be dependent on the gene. A score = 0 is equivalent to a gene that is not essential, whereas a score of -1 corresponds to the median of all common essential genes.

Due to conflicting results between the ONCOMINE and GEPIA data, we further studied the dependency of AML cells on GPX4 and AIFM2. The DepMap assay illustrated that AML cells significantly relied on GPX4 (Figure 3A, Supplementary Figure 1A) and AIFM2 (Figure 3B, Supplementary Figure 1B), with a particularly notable dependence on GPX4.

### The prognostic significance of GPX4 and AIFM2 in AML

**Figure 4:**
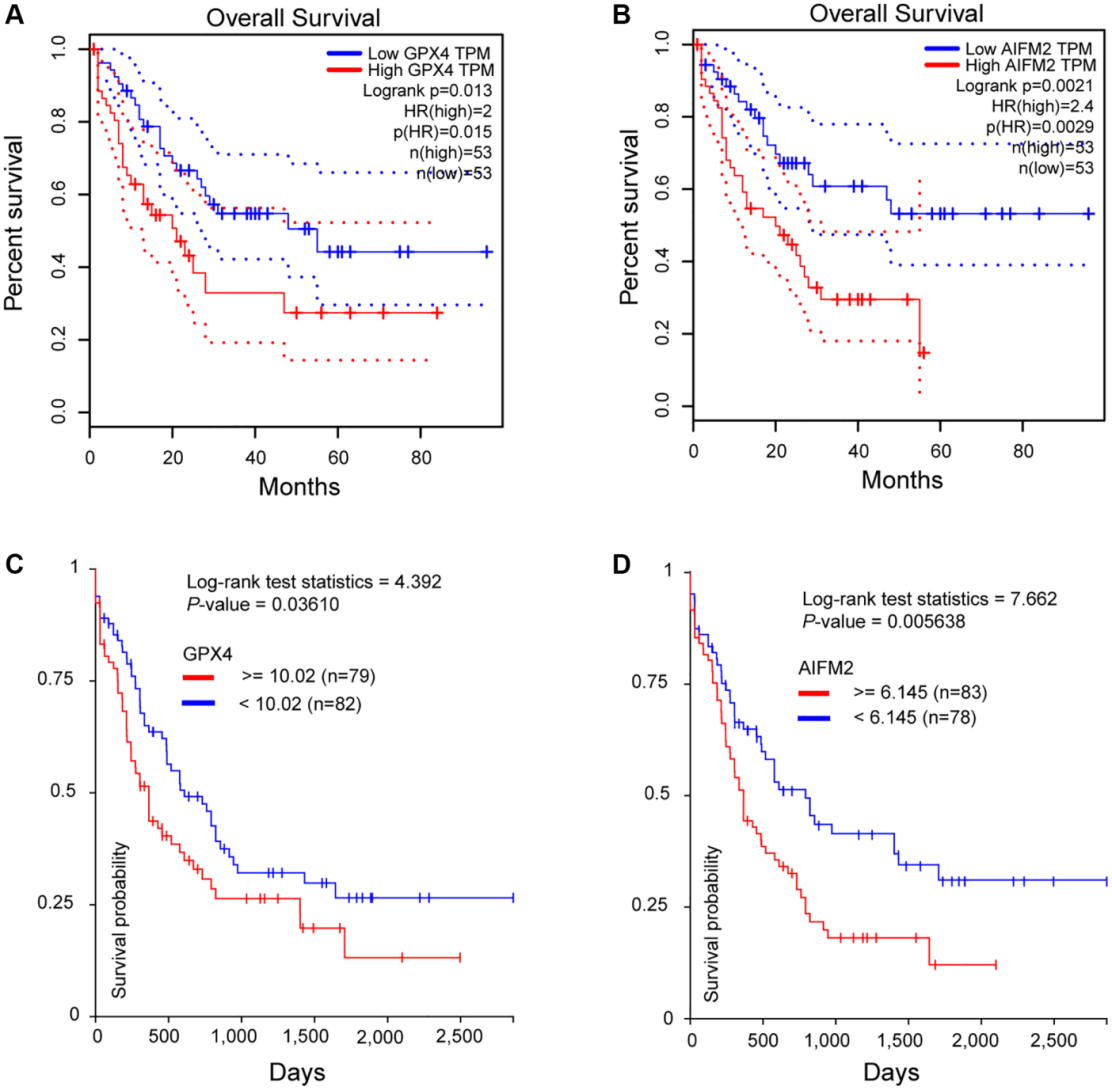
**The prognostic significance of GPX4 and AIFM2 in AML patients.** (**A**, **B**) were analyzed in the GEPIA database, and (**C**, **D**) were obtained via the UCSC Xena platform.

To assess the significance of GPX4 and AIFM2 in the survival of AML patients, we utilized the GEPIA and UCSC Xena online tools. Results from both databases indicated that increased expression of GPX4 and AIFM2 significantly correlated with poor overall survival (OS) of AML patients (Figure 4).

### Correlation between gene mutations and GPX4/AIFM2 expression

**Figure 5:**
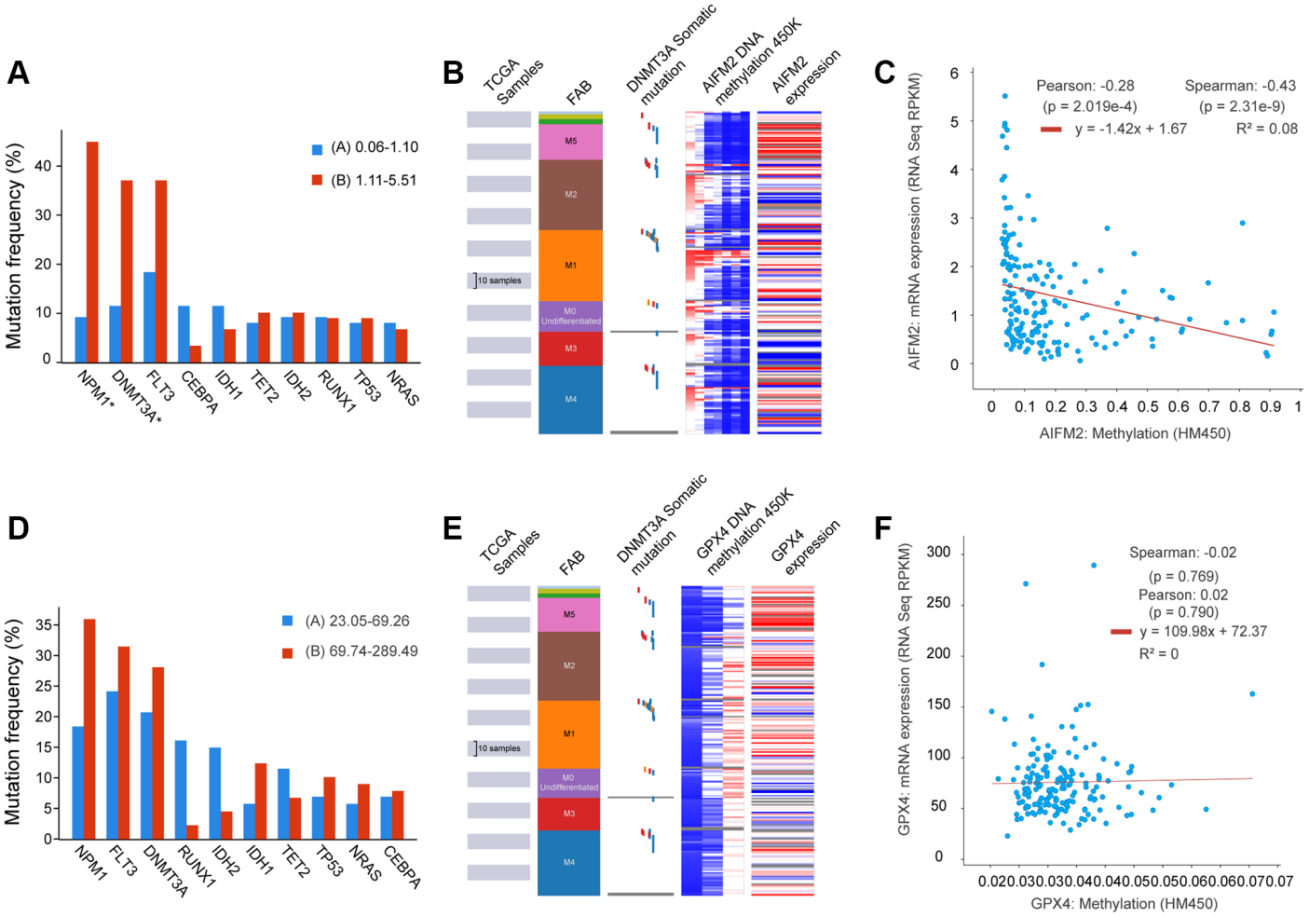
The relationship between AIFM2 expression and the mutation frequency of related genes, including NPM1, DNMT3A, FLT3, IDH1, and IDH2 (**A**). Methylation at the DNA promoters of AIFM2 in the FAB subtype (**B**). The correlation of AIFM2 expression levels and methylation (**C**). The relationship between GPX4 expression and the mutation frequency of related genes, including NPM1, DNMT3A, FLT3, IDH1, and IDH2 (**D**). Methylation at the DNA promoters of GPX4 in the FAB subtype (**E**). The correlation of GPX4 expression levels and methylation (**F**).

Given that gene mutations are prevalent in AML, we examined the correlation between GPX4 and AIFM2 transcriptional levels and gene mutations using the cBioPortal tool. Results demonstrated that AIFM2 overexpression correlated with a high mutation frequency of nucleophosmin (NPM1) and DNA methyltransferase 3A (DNMT3A) (Figure 5A). The combined use of UCSC Xena and cBioPortal tools unveiled that AML individuals with DNMT3A mutations exhibited hypomethylation of the AIFM2 promoter region and high mRNA expression (Figure 5B). Hypomethylation in the AIFM2 promoter region is inversely related to AIFM2 expression (Figure 5C). However, the GPX4 expression level showed no correlation with gene mutations or promoter methylation (Supplementary Figure 2 and Figure 5D–5F).

### Functional prediction and pathway enrichment analysis of GPX4 and AIFM2 in AML

**Figure 6:**
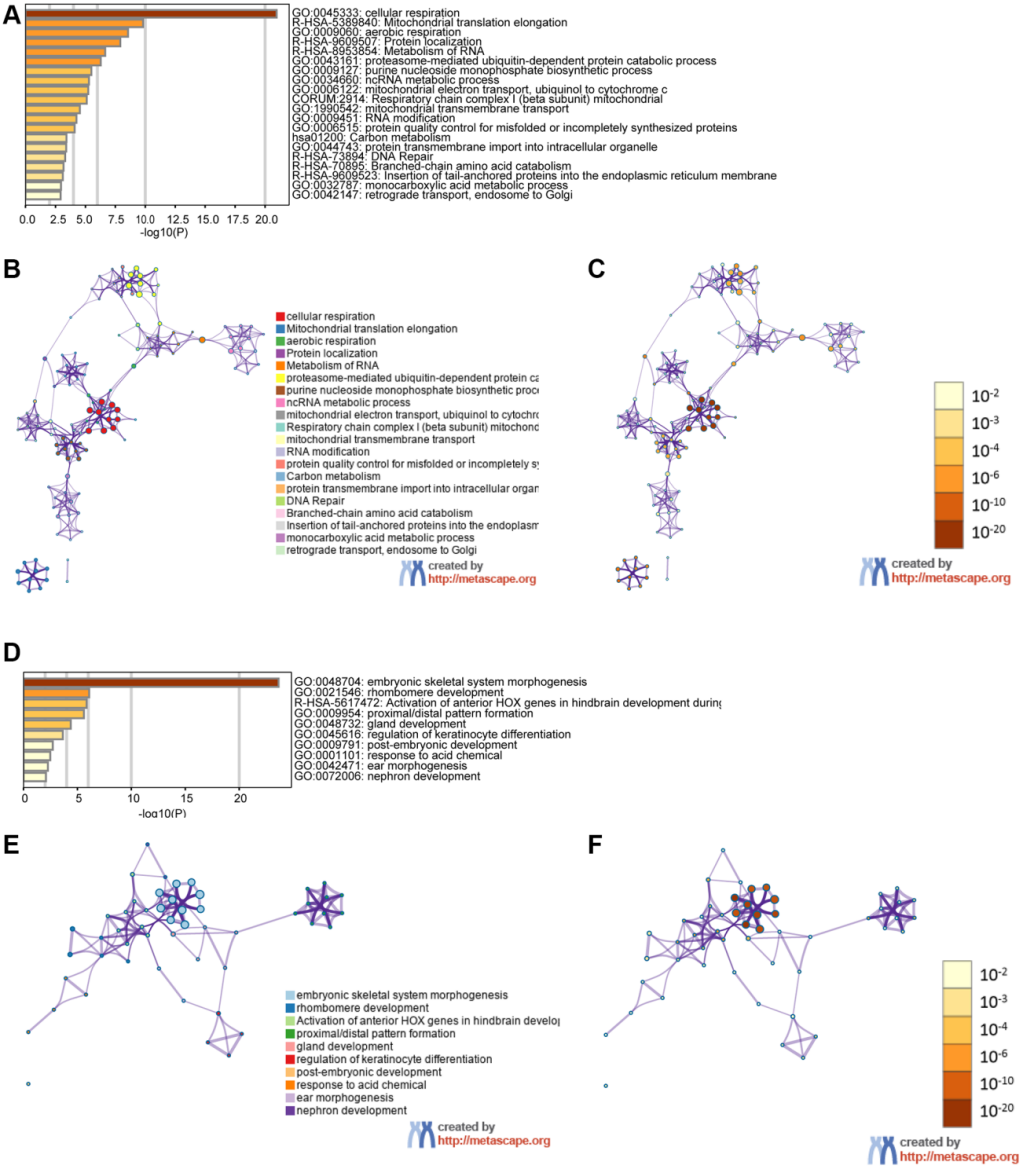
**The functions of the GPX4 and AIFM2 genes and the variant genes significantly associated with GPX4 and AIFM2.** Heat map of the GO and KEGG enriched terms colored by *P*-values. (**A**) GPX4-related genes enriched. (**B**) Network of GO and KEGG enriched terms for GPX4 colored by cluster. (**C**) Network of GO and KEGG enriched terms for GPX4 colored by *P*-value. (**D**) AIFM2-related genes enriched. (**E**) Network of GO and KEGG enriched terms for AIFM2 colored by cluster. (**F**) Network of GO and KEGG enriched terms for AIFM2 colored by *P*-value.

We examined genes coexpressed with GPX4 and AIFM2 in the TCGA-AML dataset using the cBioPortal tool. The expression of GPX4 exhibited a positive correlation with the upregulation of several genes, including POLR2E, ATP5F1D, NDUFS8, NAA10, PLEKHJ1, C19ORF24, CIAO2B, NDUFB7, SIRT6, MRPS12, UQCR11, C19ORF53, CCDC124, TBCB, and NDUFS6. Similarly, AIFM2 expression was positively correlated with elevated levels of genes such as ABHD11, LINC00899, HOXA10, HOXA6, HOXA3, HOXA7, H2AFY2, CPNE8, RINL, HOXA4, ITM2A, HOXA9, HOXA11, HOXA5, and SHISA4. Subsequently, two lists of the most frequently coexpressed genes, one list of genes related to GPX4 and another related to AIFM2, were compiled to show the results of the pathway enrichment assays using GO and KEGG tools in Metascape. The results illustrated that GPX4 affected the following processes: GO:0045333, cellular respiration; R-HSA-5389840, mitochondrial translation elongation; GO:0009060, aerobic respiration; R-HSA-9609507, protein localization; R-HSA-8953854, metabolism of RNA, GO:0043161, proteasome-mediated ubiquitin-dependent protein catabolic process; GO:0009127, purine nucleoside monophosphate biosynthetic process; GO:0034660, NcRNA metabolic process; GO:0006122, mitochondrial electron transport from ubiquinol to cytochrome c; CORUM:2914, respiratory chain complex I (beta subunit) mitochondria; GO:1990542, mitochondrial transmembrane transport; GO:0009451, RNA modification; GO:0006515, protein quality control for misfolded or incompletely synthesized protein; hsa01200, carbon metabolism; GO:0044743, protein transmembrane import into intracellular organelle; R-HSA-73894, DNA repair; R-HSA-70895, branched-chain amino acid catabolism; R-HSA-9609523, insertion of tail-anchored proteins into the endoplasmic reticulum membrane; GO:0032787, monocarboxylic acid metabolic process; and GO:0042147, retrograde transport from endosomes to Golgi (Figure 6A–6C). AIFM2 gene alterations influenced the following pathways: GO:0048704, embryonic skeletal system morphogenesis; GO:0021546, rhombomere developmental R-HSA-5617472, activation of anterior HOX genes in hindbrain development during early embryogenesis; GO:0009954, proximal/distal pattern formation; GO:0048732, gland development; GO:0045616, regulation of keratinocyte differentiation; GO:0009791, postembryonic development; GO:0001101, response to acid chemical; GO:0042471, ear morphogenesis; and GO:0072006, nephron development (Figure 6D–6F).

## DISCUSSION

Cancer cells with dysregulated ROS production, altered iron levels, and increased lipid peroxidation are more susceptible to ferroptosis, a phenomenon that has garnered significant attention in recent decades. While studies across various cancers have highlighted the efficacy of ferroptosis induction, particularly by inhibiting GPX4 and AIFM2, in eliminating apoptosis-resistant cancer cells, the exploration of ferroptosis in the context of AML remains relatively limited. In this study, we conducted a bioinformatics analysis to assess ferroptosis inducibility in AML patients. The findings revealed that high expression levels of both GPX4 and AIFM2 are associated with an adverse prognosis, suggesting the potential therapeutic use of ferroptosis inducers in treating AML patients.

Intracellular iron should be tightly controlled, and iron overload induces the overproduction of ROS through the “Fenton reaction” and “Haber-Weiss reaction” [29, 30]. ROS are regarded as double-edged swords and are the main contributors to oxidative stress. Low levels of ROS function as vital second messengers, but at high concentrations, they are cytotoxic agents in both cancer cells and healthy cells [31]. Elevated ROS reduces the differentiation and self-renewal capacity of hematopoietic stem cells (HSCs), resulting in rapid bone marrow failure for which there is no successful treatment [32, 33]. In addition, ROS participates in multilevel biological behaviors through the oxidation of critical cysteine residues in signaling proteins to drive leukemogenesis in AML [34].

Excessive production of extracellular ROS (mean > 10-fold change) was observed in >60% of primary AML blasts, and this change was not correlated with the molecular subtype of AML. These accumulated ROS promote the proliferation of AML cell lines and primary AML blasts but promote normal CD34+ cell growth to a lesser extent [35]. Interestingly, studies have also shown that ROS overproduction is related to molecular mutations, including Fms-like tyrosine kinase 3 internal tandem duplications (FLT3-ITDs), in NPM1, isocitrate dehydrogenase (IDH), and DNMT3A.

FLT3 mutation occurs in almost 30–35% of AML, and FLT3-ITD is considered closely related to increased ROS production in AML [36, 37]. For example, ROS overproduction was observed in the primary MV4-11 and MOLM-13 AML cell lines containing FLT3-ITD mutation compared to the level in the wild-type FLT3 cell line. In the mouse-derived 32D and Ba/F3 hematopoietic progenitor cell lines, which are transduced to stably express human FLT3-ITD or FLT3-TKD (D835Y) mutations, high levels of endogenous ROS are produced and result in higher oxidative DNA damage compared to that in untransfected cells [37, 38]. AML patients with FLT3-ITD have lower levels of ROS in the condition of coexisting NPM1 mutations, which occur in up to 30% of AML cases [39, 40]. This information may provide insights into the unfavorable prognosis associated with AML patients with FLT3-ITD mutations and wild-type NPM1 compared to those with both NPM1 and FLT3-ITD mutations [41]. The clinical outcomes align with evidence of increased DNA damage, suggesting a mechanistic link between FLT3-mutant AML, genomic instability, and lipid peroxidation through elevated ROS production.

IDH1 and IDH2 mutations are identified in nearly 10-15% of AML cases [42, 43]. These mutations lead to the production of R-2-hydroxyglutarate (R-2-HG), an oncometabolite that interferes with myeloid differentiation through epigenetic modifications [44]. Additionally, accumulated R-2-HG has been shown to increase intracellular ROS levels, phosphorylate NF-κB, and stimulate the proliferation of IDH-mutated AML cells via an extracellular signal-regulated kinase-dependent pathway [45].

DNMT3A mutations, found in approximately 20% of AML cases, particularly in M4-AML and M5-AML, result in the dysfunction of DNMT3A protein and alterations in DNA methylation patterns [46, 47]. Notably, hypomethylation in the AIFM2 promoter region, compared with the GPX4 promoter, leads to high AIFM2 expression in AML patients with DNMT3A mutations. This novel finding suggests a distinctive mode of ferroptosis regulation by AIFM2 in the context of DNMT3A mutation (see Supplementary Figure 3). Reports indicate that introducing the DNMT3A-Arg 882His/Cys mutant into U937 cells decreases ROS levels [48]. However, further validation is required, especially considering the unique characteristics of U937 cells derived from the pleural fluid of lymphoma patients, which may not fully represent AML characteristics.

Iron overload contributes to cellular dysfunction by upregulating ROS levels in AML cells. Interestingly, high levels of both GPX4 and AIFM2 protect AML cells from ferroptosis, underscoring the dependency of AML cells on GPX4 and AIFM2 for survival. Moreover, AML patients exhibiting elevated levels of either GPX4 or AIFM2 experienced worse overall survival (OS) compared to those with lower levels of either GPX4 or AIFM2.

These findings provide valuable insights into the intricate interplay between genetic mutations, ROS regulation, and the roles of GPX4 and AIFM2 in AML pathogenesis and prognosis.

Metabolomics studies have revealed that AML cells exhibit an increased demand for glucose and glutamine, channeling these resources to sustain proliferation advantages compared to normal cells [49, 50]. Addiction to glucose produces more α-ketoglutaric acid (α-KG), an intermediate product of the mitochondrial tricarboxylic acid cycle, converted to glutamate by aminotransferase [51]. Increased uptake of glutamine shunts more glutamine into the glutaminolysis pathway to produce glutamate through glutaminase 2 (GLS2) [52]. Accumulated glutamate facilitates the oxidized form of cysteine and cystine exchange from the extracellular space by system X_c_^-^ [53]. Overall, high concentrations of glucose and glutamine lead to abundant glutamate and cystine, which facilitates the synthesis of GSH, an essential cofactor of GPX4, to counteract ferroptosis in AML cells. Therefore, targeting key genes involved with ferroptosis cofactors should also be considered, as GPX4 inhibition alone has only modest effects on AML [54].

Certain ferroptosis inducers (FIs) have entered clinical trials and have shown promise in hematological malignancies. System X_c_^-^ inhibition, for instance, effectively suppresses tumor growth in experimental models of fibrosarcoma and diffuse large B-cell lymphoma. Depletion of GSH has been found to increase the survival of chronic lymphocytic leukemia model mice [55–58]. In addition, the FLT3 inhibitor sorafenib has also been validated as a useful FI that significantly improves the survival of patients with FLT3-ITD [59, 60]. This outcome may be due to sorafenib not only being an inhibitor of FLT3 but also a prohibitor of system X_c_^-^, thereby inducing the ferroptosis of AML cells [61]. In addition, all-trans retinoic acid derivatives also induce ferroptosis and the differentiation of AML cells through the NRF2 pathway [62]. However, it’s noteworthy that there are limited studies targeting GPX4 and AIFM2 in hematological malignancies to date.

These findings underscore the potential of targeting ferroptosis pathways and associated cofactors as a therapeutic strategy in AML, opening avenues for further exploration and development of novel treatments.

In this study, the utilization of GO and KEGG online analysis tools allowed us to elucidate the intricate association patterns between GPX4, AIFM2, and their most frequently altered linked genes in AML initiation and prognosis. Several enriched pathways emerged as noteworthy for future investigations, including GO:0045333, cellular respiration, R-HSA-5389840, mitochondrial translation elongation; GO:0009060, aerobic respiration; R-HSA-9609507, protein localization; R-HSA-8953854, metabolism of RNA; GO:0048704, embryonic skeletal system morphogenesis; and R-HSA-5617472, activation of anterior HOX genes in hindbrain development during early embryogenesis. Among these frequently altered correlated genes, R-HSA-8953854, related to the metabolism of RNA, inspired us to discover the correlation between RNA stability and ferroptosis.

The apparent paradox observed in the GEPIA and ONCOMINE studies may be attributed to the different acquisition sites of normal samples in the GTEx database and AML patients in the TCGA database. Healthy control samples in the GTEx database are derived from bone marrow, the body’s most abundant source of iron. This suggests that hematopoietic stem cells (HSCs) and differentiated cells in bone marrow must upregulate GPX4 and AIFM2 to protect themselves against ferroptosis under iron overload conditions, maintaining normal hematopoietic function. On the other hand, samples from AML patients in the TCGA dataset were obtained from peripheral blood, an environment with lower iron levels. This seeming paradox prompted consideration for using a uniform sample type to more accurately evaluate the inducibility of ferroptosis.

## CONCLUSION

The challenge of chemo-drug resistance in traditional AML treatment has prompted the exploration of novel cell death mechanisms. Ferroptosis, a recently observed form of cell death, holds promise for overcoming chemo-drug resistance and eliminating resilient cancer cells. This study delves into the correlation between the expression of key genes involved in ferroptosis and the occurrence and progression of AML. The elevated expression of GPX4 and AIFM2 emerges as a potential indicator, suggesting their utility as biomarkers for predicting poor prognosis in AML patients. These genes not only serve as prognostic biomarkers but also present themselves as potential targets for treatment in AML. The induction of ferroptosis, combined with traditional treatments, may pave the way for improved stratified therapy in AML. In conclusion, the exploration of ferroptosis in the context of AML opens avenues for innovative therapeutic strategies, offering hope for enhanced treatment outcomes and a step forward in overcoming drug resistance challenges.

## MATERIALS AND METHODS

### Cancer dependency map

The Cancer Dependency Map portal (DepMap) (https://depmap.org/portal/) is a processor used to identify essential genes across cancer cell lines by genome-wide CRISPR and shRNA screening, DepMap has already been validated as a powerful tool useful for discovering the genetic vulnerabilities of cancer cells and identifying potential targets for candidate drug screening. For a candidate gene, the calculated “perturbation score” represents the degree to which its loss prohibits cell proliferation in a selected cell line. A lower score (<0) means that the candidate gene is more likely to be required in the selected cell line. A score = 0 is equivalent to a gene that is not essential whereas a score of −1 corresponds to the median of all common essential genes. We used the DepMap portal to assay the dependence of AML cell lines on GPX4 and AIFM2.

### Functional enrichment and bioinformatics analysis

The Metascape portal (http://metascape.org) provides an online assay tool, that covers more than 40 independent knowledge bases in an integrated database including functional enrichment, interactome analysis, gene annotation, and a membership search in combination with Gene Ontology (GO) and the Kyoto Encyclopedia of Genes and Genomes (KEGG) tools within Metascape [63]. Genes with a correlation value > 0.5 were selected for pathway enrichment. We generated two lists of enriched genes associated with GPX4 and AIFM2 respectively, to identify the most frequently altered linked genes.

### Gene expression profiling interactive analysis (GEPIA) dataset

GEPIA is a web-based tool that enables researchers to perform fast and customizable assays based on TCGA and Genotype-Tissue Expression (GTEx) data [64]. Using GEPIA, we validated the expression and prognostic significance of GPX4 and AIFM2 in AML patients.

### ONCOMINE database analyses

The ONCOMINE database serves as an online analysis tool that enables users to assay the transcriptional level of genes of interest in different cancer types based on a microarray database [65]. GPX4 and AIFM2 gene transcription levels were analyzed in multiple cancer types using a gene expression array within ONCOMINE. GPX4 and AIFM2 mRNA expression levels in tumor specimens were compared with those of a normal control. The cutoff *P* value was defined as 0.05, the fold change was defined as all.

### UCSC Xena and cBioPortal tools

The UCSC Xena (http://xena.ucsc.edu/) is regarded as a visual integration and exploration tool for use with public and private multiomics datasets. The Xena browser enables researchers to explore data across multiple Xena hubs using a variety of visualization types and analyses [66]. We used UCSC Xena to assay the methylation in the promotor region of GPX4 and AIFM2 as indicated in The Cancer Genome Atlas (TCGA) AML dataset. The cBioPortal (http://www.cbioportal.org/) is designed for the interactive exploration of multidimensional cancer genomics databases. It supports and stores data that include mutations, copy number alterations, mRNA expression changes, and DNA methylation values, as well as clinical parameters. The online analyses tool in cBioPortal allows researchers to interrogate datasets across genes, samples, and data types, allowing them to examine several different biologically and/or clinically relevant hypotheses. Ferroptosis-related gene mRNA expression analyses in adult patients with the FAB subtype of AML and in AML cell lines were conducted by cBioPortal [67]. Moreover, the expression levels of GPX4 and AIFM2 in the AML cell lines were also evaluated with the European Bioinformatic Institute (EMBL-EBI) database (https://www.ebi.ac.uk/gxa/home), and the expression values were log-transformed and a heat map was generated using GraphPad Prism 7.0.

### Data availability statement

The data that support the findings of this study are available in open public database. These data were derived from the following resources available in the public domain: Cbioportal at https://www.cbioportal.org/, UCSC Xena at https://xenabrowser.net/.

## Supplementary Materials

Supplementary Figures
