# Supplementary material for: The ferroptosis landscape in acute myeloid leukemia
Source: Aging (Albany NY). 2023 Nov 29;15(22):13486–503. doi: 10.18632/aging.205257 (PMC10713399; doi:10.18632/aging.205257)
Supplement: Supplementary Figures [file aging-15-205257-s001.pdf]

SUPPLEMENTARY FIGURES

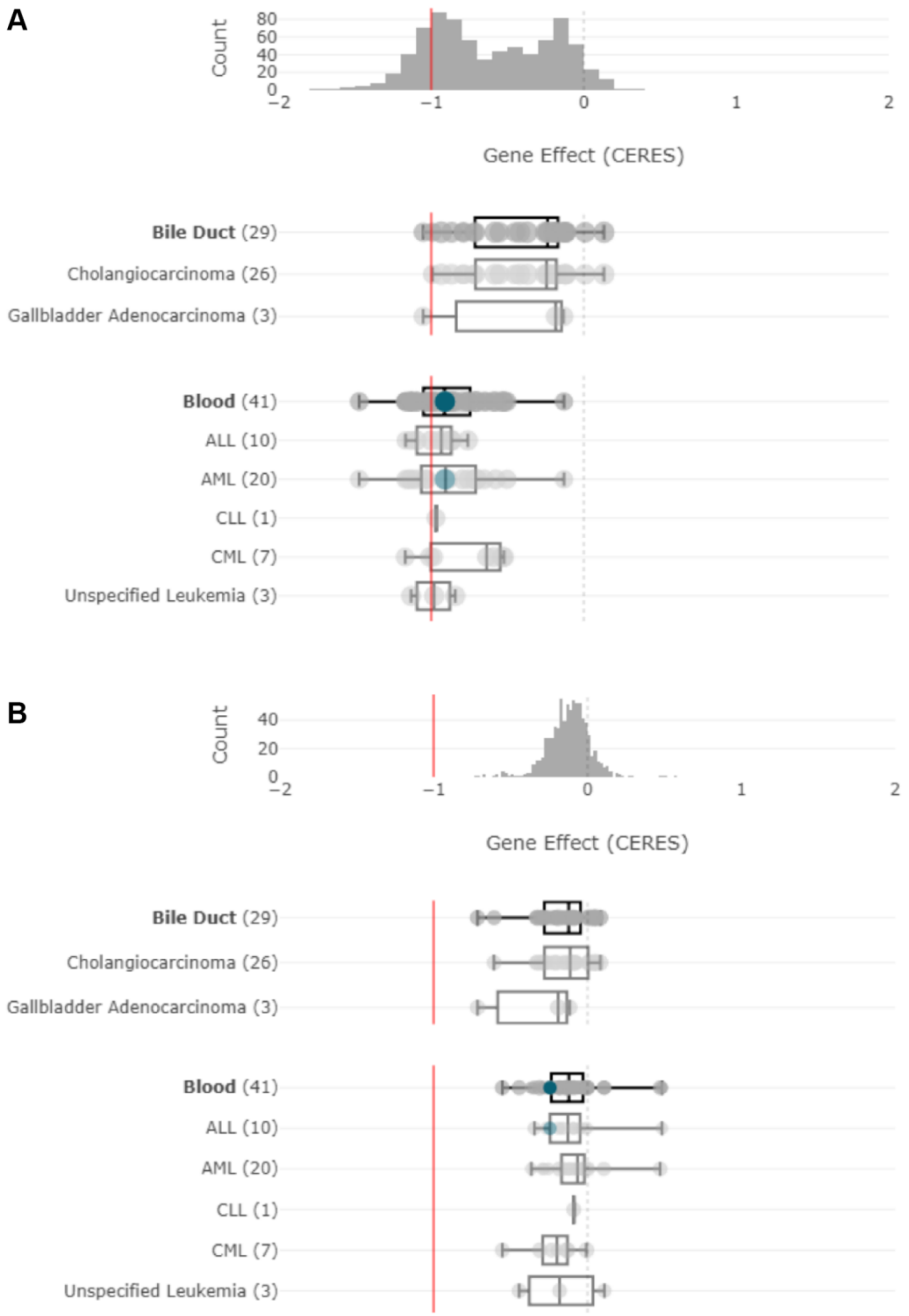

Supplementary Figure 1. The dependency of leukemia cells on GPX4 (**A**) and AIFM2 (**B**), figures are obtained from Depmap and screenshotted.

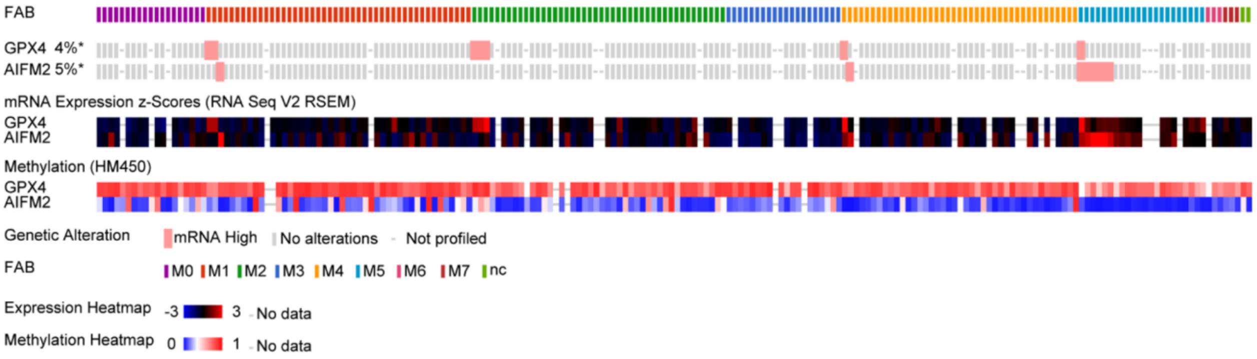

Supplementary Figure 2. Expression of GPX4 and AIFM2 in AML patients and the methylation profile of promotors in AML FAB subtype.

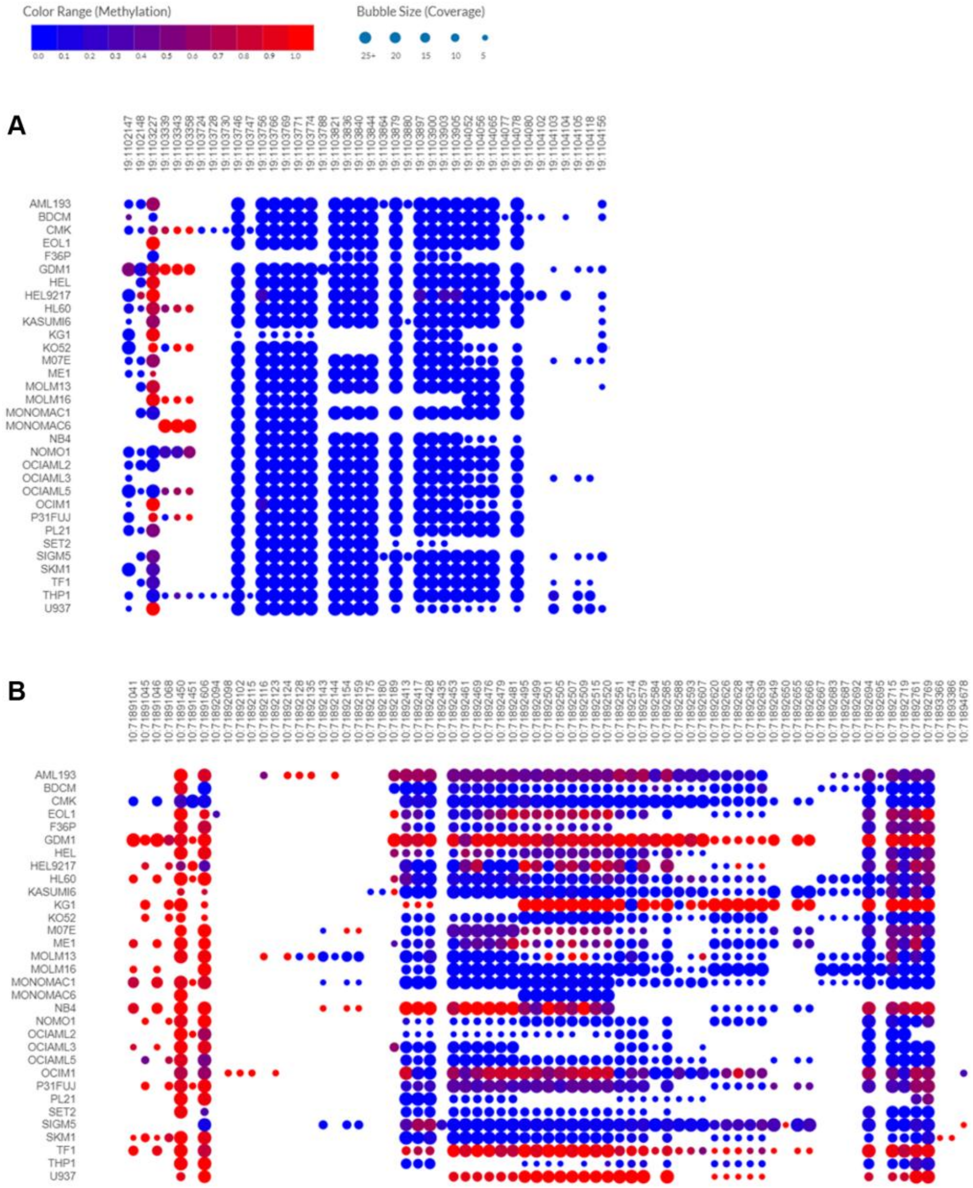

Supplementary Figure 3. CpG island methylation of GPX4 (**A**) and AIFM2 (**B**) promotors, figures are obtained from Depmap.
